# Establishment of an In Vitro Transport Assay That Reveals Mechanistic Differences in Cytosolic Events Controlling Cholera Toxin and T-Cell Receptor α Retro-Translocation

**DOI:** 10.1371/journal.pone.0075801

**Published:** 2013-10-11

**Authors:** Paul Moore, Kaiyu He, Billy Tsai

**Affiliations:** Department of Cell and Developmental Biology, University of Michigan Medical School, Ann Arbor, Michigan, United States of America; University of Toronto, Canada

## Abstract

Following retrograde trafficking to the endoplasmic reticulum (ER), cholera toxin A1 (CTA1) subunit hijacks ER-associated degradation (ERAD) machinery and retro-translocates into the cytosol to induce toxicity. We previously established a cell-based in vivo assay to identify ER components that regulate this process. However, elucidating cytosolic events that govern CTA1 retro-translocation using this assay is difficult as manipulating cytosolic factors often perturbs toxin retrograde transport to the ER. To circumvent this problem, we developed an in vitro assay in semi-permeabilized cells that directly monitors CTA1 release from the ER into the cytosol. We demonstrate CTA1 is released into the cytosol as a folded molecule in a p97- and proteasome-independent manner. Release nonetheless involves a GTP-dependent reaction. Upon extending this assay to the canonical ERAD substrate T-cell receptor α (TCRα), we found the receptor is unfolded when released into the cytosol and degraded by membrane-associated proteasome. In this reaction, p97 initially extracts TCRα from the ER membrane, followed by TCRα discharge into the cytosol that requires additional energy-dependent cytosolic activities. Our results reveal mechanistic insights into cytosolic events controlling CTA1 and TCRα retro-translocation, and provide a reliable tool to further probe this process.

## Introduction

To cause disease, cholera toxin (CT) binds to ganglioside GM1 receptor on the plasma membrane of host cells, becomes endocytosed within vesicles, and traffics through the Golgi apparatus en route to the endoplasmic reticulum (ER). Although the detailed mechanism that sorts CT from the cell surface to the ER remains to be fully clarified [1], this pathway is likely regulated by a combination of cellular lipid [2], [3] and proteinaceous [4]–[6] components. A host cell protease proteolytically cleaves the catalytic A subunit (CTA) into the CTA1 and CTA2 peptides before the toxin reaches the ER [7]. However, CTA remains as a single polypeptide chain after cleavage because of a disulfide bond that links CTA1 and CTA2. In the ER, subsequent reduction of this disulfide bond by oxidoreductases generates free CTA1 peptide. CTA1 is thought to disguise as a misfolded protein and engages the endogenous ER-associated degradation (ERAD) machinery that normally recognizes and retro-translocates misfolded proteins to the cytosol for ubiquitin-dependent proteasomal degradation [8], [9]. CTA1, however, evades this degradative fate in the cytosol [10]. Instead, it activates a signal transduction cascade that leads to chloride ion and water secretion across the plasma membrane, resulting in massive diarrhea that typifies the cholera disease.

How CTA1 is released from the ER membrane into the cytosol and escapes proteasomal destruction is not entirely clear. In the case of cellular ERAD substrates destined for the proteasome, ATP hydrolysis by the cytosolic AAA p97 ATPase (Cdc48 in yeast) is thought to drive release into the cytosol [9], [11]. The released substrate is subsequently delivered to the proteasome, potentially by the recently identified BAG6 chaperone complex [12]. The observation that CTA1 is not degraded by the proteasome [10], however, raises the question of whether p97 extracts CTA1 from the ER membrane. Two independent studies addressed this question by expressing mutant forms of p97 and reached opposite conclusions [13], [14]. Interpretation of these findings is further confounded by the fact that p97 is known to regulate a myriad of cellular functions [15], [16], including endocytosis [17], [18], Golgi and ER biogenesis [19], and ER fusion [20]. Hence, the direct role of p97 in cytosolic release of CTA1 remains unclear.

We previously established a cell-based in vivo assay designed to analyze the role of ER-resident lumenal and membrane components that prime CTA1 for retro-translocation into the cytosol [21]. In this assay, CT-intoxicated cells are fractionated following gentle detergent treatment to generate two pools representing either cytosol- or membrane-localized toxin. Correlating the activity or expression of a defined ER-resident protein with levels of cytosol-localized toxin reveals any potential function of the targeted ER factor in controlling ER-to-cytosol transport of CTA1. This assay has since been used by many laboratories to examine CTA1 retro-translocation [22]–[24], and ER membrane penetration of a DNA tumor virus [25]–[27]. However, this assay is not ideally suited to examine cytosolic processes regulating CTA1 retro-translocation: disrupting cytosolic factors could unintentionally affect transport of CT from the plasma membrane to the ER, as may be the case for perturbing p97.

To circumvent this problem, we modified our cell-based in vivo assay and established an in vitro assay. Cytosolic extract (CE) is incubated with a semi-permeabilized membrane fraction containing ER-localized CTA1 and induces release of the toxin from the membrane. This reaction mimics toxin release from the ER into the cytosol in intact cells. The unique advantage of this assay is that any cytosolic protein hypothesized to catalyze the release reaction can be manipulated directly in the context of the CE without disrupting the integrity of the membrane fraction. We then adapted this assay to examine cytosolic events controlling retro-translocation of the canonical ERAD substrate T-cell receptor α (TCRα), a type I transmembrane glycoprotein. Our findings reveal significant mechanistic differences in cytosolic events guiding retro-translocation of CTA1 and TCRα. Importantly, they also suggest that uncharacterized cytosolic GTPases eject CTA1 into the cytosol. Establishing this assay now affords an opportunity to more accurately elucidate cytosolic events governing ER-to-cytosol transport of pathogens and misfolded cellular proteins.

## Materials and Methods

### Materials

Polyclonal His, Hsp90, and PDI antibodies were purchased from Santa Cruz Biotechnology (Santa Cruz, CA); monoclonal Hsc/p70, p97/VCP, PDI, and polyclonal GRP78/BiP and CTB antibodies from Abcam (Cambridge, MA); polyclonal S8/PA700 antibody from Affinity Bioreagents (subsidiary of Thermo Fisher Scientific, Waltham, MA); and polyclonal ERGIC-53/p58 antibody from Sigma-Aldrich (St. Louis, MO). Monoclonal antibodies against HA and Hsp27 were gifts from K. Verhey (University of Michigan) and M. Welsh (University of Michigan), respectively. Polyclonal CTA antibody was produced against denatured CTA purchased from EMD Biosciences (San Diego, CA). Purified CT was also purchased from EMD Biosciences. Purified CTA was purchased from Enzo Life Sciences (Farmingdale, NY). R192G CT was a gift from W. Lencer (Boston Children's Hospital, Boston, MA). Yeast CE was generously provided by Robert Fuller (University of Michigan). A C-terminally HA-tagged T-cell receptor alpha (TCRα-HA) construct was a gift from C. Wojcik (Indiana University). His-tagged p97 constructs (WT p97-His and p97-QQ-His) were gifts from Y. Ye (National Institutes of Health).

### Tissue culture, transfection and drug treatment

HEK 293T cells were cultured in DMEM with 10% fetal bovine serum (FBS) and penicillin/streptomycin. All expression constructs used were transfected into 30% confluent cells on 10-cm dishes using the Effectene system (Qiagen, Chatsworth, CA). Where indicated, Brefeldin A (BFA; Sigma-Aldrich, St. Louis, MO) was added to cells in complete media at 2 μg/ml one hour prior to intoxication and maintained in all subsequent media and buffers. Epoxomicin (Calbiochem, a subsidiary of EMD Millipore, Billerica, MA) was added in complete media at 0.5 μg/ml for two hours at 37°C and removed before harvesting cells. Peptide-N-glycosidase F (PNGase F) and Endoglycosidase H (Endo H) were used as according to the manufacturer instructions (New England Biolabs, Ipswich, MA).

### In vivo and in vitro retro-translocation assays

293T cells were intoxicated with 10 nM CT in HBSS for 45 minutes at 37°C. Cells (2×10^6^) were semi-permeabilized in 100 μl of 0.01% digitonin in HCN buffer (50 mM HEPES, pH 7.5, 150 mM NaCl, 2 mM CaCl_2_, and protease inhibitors), incubated on ice for 10 minutes, and centrifuged at 16,000×g for 10 minutes at 4°C to generate supernatant (S1) and pellet (P1) fractions. S1 was spun in an ultracentrifuge at 100,000×g for 30 minutes at 4°C to generate new supernatant (S1′) and pellet fractions. S1′ contains in vivo retro-translocated toxin. To generate the CE, non-intoxicated cells were permeabilized and fractionated as above. The final supernatant represents the cytosolic extract (CE). P1 was resuspended in unmodified or modified CE, incubated at room temperature for 30 minutes, and centrifuged at 16,000×g for 10 minutes at 4°C to generate secondary supernatant (S2) and pellet (P2) fractions. S2 was spun in an ultracentrifuge at 100,000×g for 30 minutes at 4°C to generate new secondary supernatant S2′ and pellet P2′ fractions. S2′ contains in vitro retro-translocated toxin. 15% of P1 and P2 fractions and 30% of S1, S1′, S2, P2′, and S2′ fractions were input for analysis by non-reducing SDS-PAGE and immunoblotting.

### Trypsin sensitivity assays

P1, S1′, S2, S2′ and CE fractions were prepared as above. CE fractions were supplemented with 2 nM purified CTA or CTA1 using 20 mM dithiothreitol (DTT; Sigma-Aldrich). Purified trypsin (Sigma-Aldrich) was added to fractions at a final concentration ranging from 0.02–0.3 mg/ml. Trypsin digestion was performed at 4°C for one hour before inactivation of the reaction with 4 mM Tosyl-Lys-chloromethylketone (TLCK) (Sigma-Aldrich). Samples were analyzed by non-reducing SDS-PAGE and immunoblotting.

### Cytosolic extract modification

Immunodepletion of p97 was accomplished by addition of a monoclonal antibody against p97 to CE and incubation at 4°C for three hours. Immune-complexes were captured by addition of protein A and protein G agarose beads (Invitrogen, Carlsbad, CA) and the resulting supernatant collected and applied to the in vitro retro-translocation assay. CE energy state was altered by addition of 1U/50 μl grade VII apyrase (Sigma-Aldrich), ATP-γ-S or GTP-γ-S (Roche, Indianapolis, IN) to a 0.01–10 mM final concentration, as indicated. Modified extracts were incubated at room temperature for 30 minutes, returned to 4°C, and then applied to the in vitro retro-translocation assay.

### Size Exclusion Chromatography

1 ml of 293T CE was generated as above and passed through a Bio-Sil SEC 250 (Bio-Rad, Hercules, CA) size exclusion column using HCN buffer for the mobile phase. Individual fractions were collected in 250 μL increments and analyzed for protein content by UV absorption, Coomassie brilliant blue staining, Bradford protein quantification, and immunoblotting. Fractions were then applied to the in vitro retro-translocation assay to determine activity. Molecular weights were estimated using Gel Filtration Standard (Bio-Rad).

### Dialysis

Samples were loaded to a microdialysis system utilizing Spectra/Por dialysis membrane with a 12–14 kDa molecular weight cut off (Spectrum Laboratories, Rancho Dominguez, CA). Samples were dialyzed 400-fold against HCN buffer for 2 hours at 4°C to remove small molecules.

*All data are representative of three or more unique experiments.*

## Results

### Establishment of an in vitro cholera toxin retro-translocation assay

**Figure 1:**
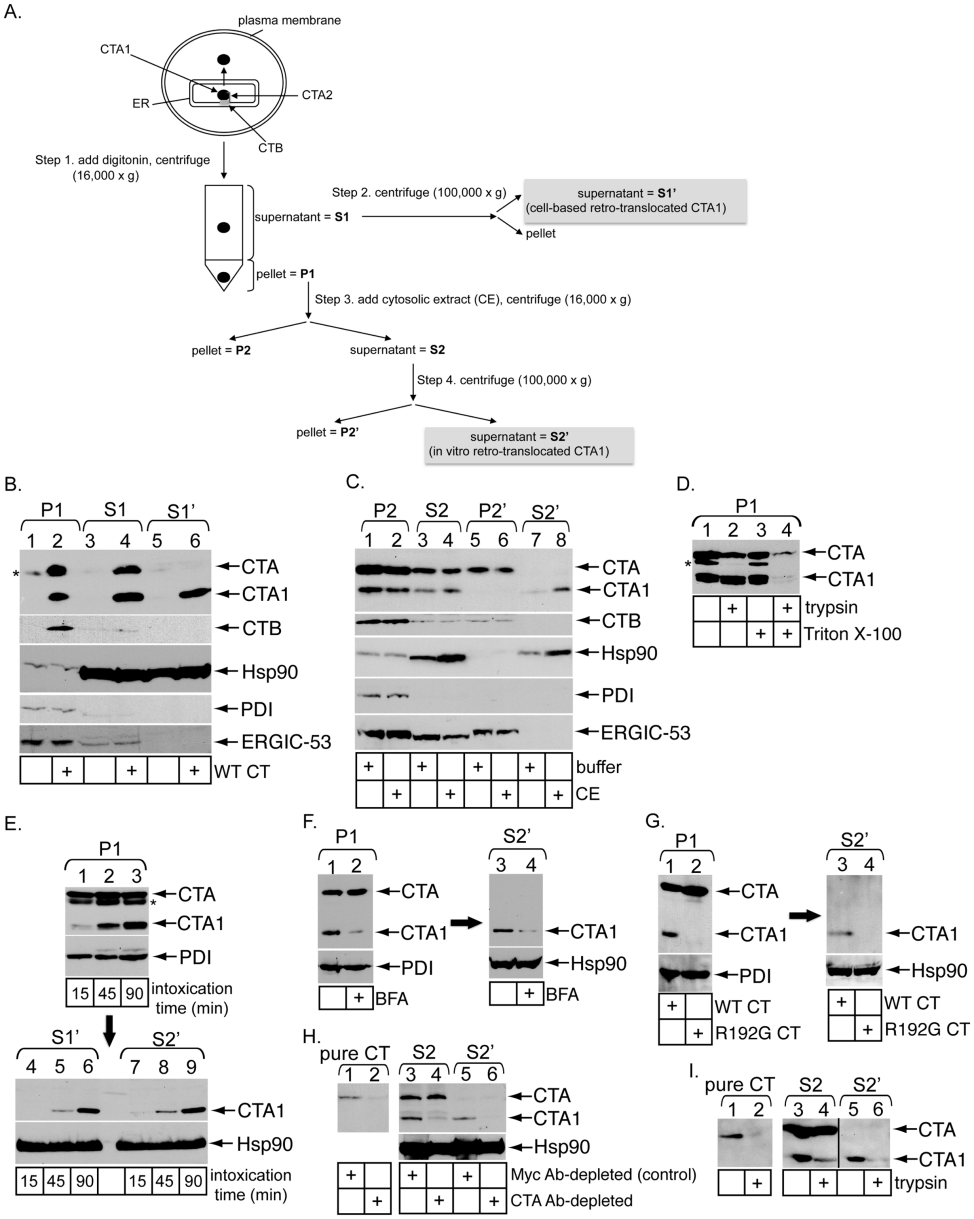
Establishment of an in vitro cholera toxin retro-translocation assay. (A) Flow diagram of the in vitro retro-translocation assay. Steps 1–4 indicate generation of unique fractions, including P1, S1, S1′, P2, S2, P2′, and S2′. (B) 293T cells were intoxicated with or without 10 nM CT for 45 minutes, permeabilized with digitonin (0.01%), and processed as described in A to generate P1, S1, and S1′. Fractions were analyzed by immunoblotting with the indicated antibodies. * denotes an unidentified protein in the P1 that cross-reacts with the CTA antibody. (C) CT-intoxicated P1 was resuspended in buffer or a cytosolic extract (CE). After incubation for 30 minutes at room temperature, samples were processed to generate P2, S2, P2′, and S2′. Fractions were analyzed by immunoblotting with the indicated antibodies. (D) CT-intoxicated P1 was resuspended in buffer with or without 1% Triton X-100 and with or without 0.3 mg/ml trypsin. Proteolysis proceeded at 4°C for one hour before reactions were stopped with TLCK and samples analyzed by immunoblotting with CTA antibody. * denotes an unidentified protein in the P1 that cross-reacts with the CTA antibody. (E) Cells were incubated at 4°C for 30 minutes in the presence of 20 nM CT to allow binding of toxin to the plasma membrane, washed to remove excess toxin, and then incubated at 37°C to synchronize toxin uptake. Intoxicated cells were harvested after 15, 45 or 90 minutes and subjected to the in vitro retro-translocation assay. P1, S1′ and S2′ fractions were analyzed by immunoblotting with the indicated antibodies. (F) Cells were treated with vehicle or BFA prior to and during intoxication. Intoxicated cells were subjected to the in vitro retro-translocation assay to generate P1 and the corresponding S2′. P1 and S2′ were analyzed by immunoblotting with the indicated antibodies. (G) Cells intoxicated with WT or R192G CT were subjected to the in vitro retro-translocation assay to generate P1 and the corresponding S2′. These fractions were analyzed by immunoblotting with the indicated antibodies. (H) Purified CTA, S2 and S2′ were incubated with the control Myc antibody or an antibody against CTA. Immune-complexes were precipitated using protein A agarose beads and the resulting supernatants analyzed by immunoblotting with the indicated antibodies. (I) Purified CTA, S2 and S2′ were incubated with buffer or 0.3 mg/ml trypsin at 4°C for one hour before reactions were stopped with TLCK and analyzed by immunoblotting with CTA antibody.

To establish an in vitro toxin retro-translocation assay, we first demonstrated the integrity of a modified version of our established cell-based in vivo retro-translocation assay [21]. 293T cells intoxicated with or without CT were treated with a low digitonin concentration followed by medium speed centrifugation (16,000× g) of the sample to generate pellet (P1) and supernatant (S1) fractions (Figure 1A, step 1). P1 should harbor large intact membranes including the plasma and ER membranes, while S1 should contain cytosolic proteins as well as small budded vesicles and fragmented membranes that did not pellet under the medium speed spin. The S1 is further subjected to a high speed spin (100,000× g) to generate a new supernatant fraction called S1′ that is devoid of vesicles and fragmented membranes (Figure 1A, step 2). As anticipated, the soluble cytosolic marker Hsp90 was found mostly in S1 and S1′ (Figure 1B, third panel, compare lanes 3–6 to 1 and 2), while the ER marker protein disulfide isomerase (PDI) was found only in P1 (Figure 1B, fourth panel, compare lanes 1 and 2 to 3–6). Similar results were observed using the cytosolic marker Hsp27 and the ER marker BiP (Figure S1A, compare lanes 1 and 2 to 3–6). ERGIC-53, a transmembrane lectin that cycles between the ER and the ER-to-Golgi intermediate compartment, is found constitutively in transport vesicles. This protein partitions only to P1 and S1 (Figure 1B, fifth panel, compare lanes 1–4 to 5 and 6), demonstrating that S1′ is free of membrane vesicles. These findings verify the integrity of our fractionation procedure. When cells were intoxicated with wild-type (WT) CT and subjected to the fractionation procedure, we found CTA, CTA1 and the receptor binding subunit of CT, CTB, in P1 and S1 (Figure 1B, top panel, lane 2 and 4). However, only CTA1 is present in S1′ (Figure 1B, top panel, lane 6). The presence of CTA and CTB in S1 likely reflects association with vesicles (see below) that are removed during the high speed spin. These data are consistent with the premise that only CTA1 undergoes retro-translocation to reach the cytosol. Thus, CTA1 in S1′ represents cell-based retro-translocated toxin (Figure 1A).

The CTA1 pool in P1 represents ER-localized toxin primed for release into the cytosol. If CTA1 relies on a cytosolic factor to complete retro-translocation, then a cytosolic extract (CE) should promote release of toxin exposed on the cytosolic side of the ER membrane. To test this possibility, intoxicated P1 was incubated with a control buffer or a naïve CE derived from 293T cells and centrifuged at medium speed to generate secondary pellet (P2) and supernatant (S2) fractions (Figure 1A, step 3). To account for de novo vesicle budding or non-specific membrane fragmentation, S2 was subsequently centrifuged at high speed to generate final pellet (P2′) and supernatant (S2′) fractions (Figure 1A, step 4). Similar to S1′, we found Hsp90 and Hsp27 but not CTA, CTB, PDI, BiP nor ERGIC-53 in S2′ (Figure 1C, second through fifth panels, compare lanes 7 and 8 to 1–6; Figure S1B, compare lanes 7 and 8 to 1–6). Strikingly, CTA1, but not CTA, appeared in S2′ (Figure 1C, top panel, compare lanes 7 and 8 to 1–6). Because CTA1 in P1 is protected against proteolysis in the absence of detergent (Figure 1D, compare lane 2 to 1 and 4 to 3), CTA1 in S2′ is not due to non-specific toxin leakage. Moreover, CTA1 release from the pellet fraction occurs in a CE-dependent manner (Figure 1C, top panel, compare lane 8 to 7). As generation of S2′ is analogous to S1′ formation, we conclude CE-induced release of CTA1 represents in vitro retro-translocated toxin (Figure 1A).

Prior to retro-translocation, CTA is proteolytically cleaved by a host protease [7] and traffics to the ER. Moreover, reduction of CTA to CTA1 does not occur until the toxin has trafficked through the endosomal transport pathway and trans-Golgi network to reach the ER [28]. To verify CE-induced CTA1 release depends on proper arrival of the toxin to the ER, cells were synchronously intoxicated for 15, 45 or 90 minutes and then used to generate P1, S1′ and S2′ fractions. Time-dependent generation of CTA1 in P1 demonstrates that a significant fraction of the toxin does not arrive in the ER until after 15 minutes (Figure 1E, top panel, compare lanes 1–3). More importantly, appearance of CTA1 in both S1′ and S2′ fractions coincides with ER arrival and reduction (Figure 1E, bottom panels, compare lanes 4–6 and 7–9), suggesting that both cell-based and in vitro retro-translocated CTA1 are derived from the ER.

It was previously demonstrated that ER arrival and reduction of the toxin are antogonized by treatment with brefeldin A (BFA), a drug that inhibits COPI-dependent retrograde transport from the cell surface to the ER [28]. To further establish that in vitro retro-translocation of CTA1 occurs from the ER, P1 was generated from CT-intoxicated cells pretreated with or without BFA. BFA blocked efficient CTA1 formation in P1 (Figure 1F, top panel, compare lane 2 to 1) because CT does not reach the ER and undergo reduction of CTA. CE was then incubated with control P1 or BFA-treated P1 and the CTA1 levels were measured in the resulting S2′ fraction. The reduced level of CTA1 in the S2′ derived from BFA-pretreated P1 (Figure 1F, top panel, compare lane 4 to 3) indicates that toxin arrival to the ER is a prerequisite step for CTA1 release into the cytosol in vitro.

To additionally test whether in vitro release of CTA1 resembles release in vivo, we examined an uncleavable mutant form of CTA, R192G CTA, that is not released into the cytosol in vivo [21], [29]. The R192 cleavage site is flanked by C187 and C199 that form the lone disulfide bond. Cleavage and subsequent reduction permit generation of CTA1. Hence, the non-cleavable R192G mutant does not form CTA1 even after reduction in the ER. We therefore hypothesized that, similar to the in vivo setting, CE should not stimulate release of R192G CTA into the S2′. As expected, CTA1 only appeared in P1 derived from cells intoxicated with WT CT but not R192G CT (Figure 1G, top panel, compare lane 2 to 1). Similarly, CTA1, but not R192G CTA, appeared in S2′ after incubation of the respective P1 fractions with CE (Figure 1G, top panel, compare lane 3 to 4). These findings reveal another similarity between the in vivo and in vitro toxin release reactions.

After CTA1 is released into the cytosol, the toxin should be freely soluble and not encased in vesicles. While the absence of ERGIC-53 in S2′ suggests this fraction is devoid of vesicles, this observation does not directly address whether the toxin is free and soluble. To test this possibility more rigorously, we performed two additional control experiments. First, free toxin should interact with an antibody against CTA. Indeed, when S2′ containing CTA1 was incubated with either a control Myc or CTA antibody, CTA1 was completely and specifically immuno-depleted by the CTA antibody (Figure 1H, top panel, compare lane 6 to 5). In comparison, CTA1 but not CTA in S2 was largely immuno-depleted by the CTA antibody (Figure 1H, top panel, compare lane 4 to 3), indicating that in this fraction CTA1 is freely soluble while CTA is likely encapsulated in a membrane vesicle. The inability to immuno-deplete CTA in S2 is not due to antibody preference for CTA1 (the CTA antibody is a polyclonal antibody directed against full length CTA) as this antibody can efficiently immuno-deplete purified CTA (Figure 1H, top panel, compare lane 2 to 1). As an alternative to immuno-depletion, a protease-sensitivity assay was employed. We found CTA1 in S2′ was sensitive to trypsin digestion (Figure 1I, compare lane 6 to 5), while only CTA1 but not CTA in S2 was significantly trypsin sensitive (Figure 1I, compare lane 4 to 3). CTA in the S2 is not intrinsically resistant to trypsin degradation as pure CTA is efficiently digested under identical conditions (Figure 1I, compare lane 2 to 1). We conclude CTA1 in S2′ is free and soluble, properties expected of a toxin released into the cytosol.

### The proteasome degrades cytosol-localized deglycosylated TCRα but not CTA1

**Figure 2:**
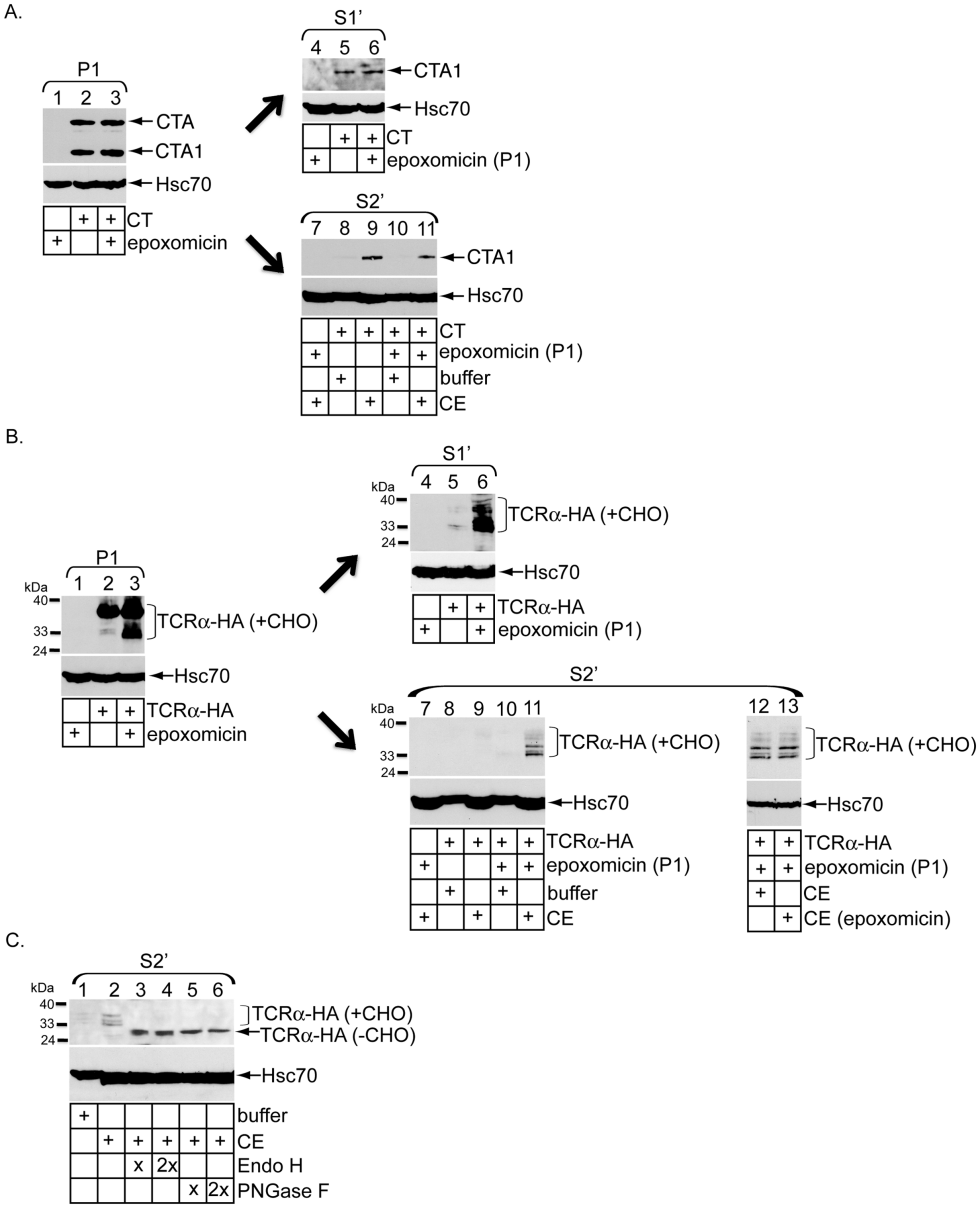
Proteasome degrades cytosol-localized deglycosylated TCRα but not CTA1. (A) 293T cells intoxicated with or without CT were co-treated with or without epoxomicin for two hours before being subjected to the in vitro retro-translocation assay. P1, S1′ and S2′ were analyzed by immunoblotting with the indicated antibodies. (B) Cells transfected with vector or TCRα-HA were treated with or without epoxomicin for two hours before permeabilization to generate P1 and S1′. P1 from cells treated with or without epoxomicin were incubated with buffer, CE, or epoxomicin-treated CE to generate S2′. Fractions were analyzed by immunoblotting with the indicated antibodies. Observed TCRα-HA species were glycosylated to various degrees (+CHO). (C) Cells transfected with TCRα-HA were subjected to the in vitro retro-translocation assay to generate S2′. Where indicated, samples were treated with either Endo H or PNGase F at two different concentrations and then analyzed by immunoblotting with the indicated antibodies. Fully deglycoslyated species are denoted as (−CHO).

Previous studies demonstrated that the proteasome neither degrades cytosol-localized CTA1 [21] nor affects toxin-induced chloride secretion in cells [10]. We therefore assessed whether the proteasome plays any role in the in vitro toxin release assay. Cells pretreated with or without the specific proteasome inhibitor epoxomicin were intoxicated with or without CT to initially generate P1 and S1′. We found epoxomicin treatment did not increase the CTA1 level in P1 (Figure 2A, top panel, compare lane 3 to 2) nor S1′ (Figure 2A, top panel, compare lane 6 to 5), demonstrating that the proteasome neither affects CT transport to the ER nor degrades CTA1 in cells. When S2′ fractions were subsequently generated, CE-induced release of CTA1 from epoxomicin-treated P1 was not increased in comparison to the toxin level derived from untreated P1 (Figure 2A, top panel, compare lane 11 to 9). Thus, in agreement with the in vivo data, the proteasome does not degrade in vitro released toxin.

Because the canonical ERAD substrate TCRα is degraded by cytosolic proteasome in cells [30], [31], we empolyed the in vitro toxin retro-translocation assay to study how TCRα is released into the cytosol. Cells transfected with or without a C-terminally HA-tagged full-length TCRα (TCRα-HA) were incubated with or without epoxomicin, and the cells processed to generate P1 and S1′. In P1, epoxomicin moderately stabilized fully glycoslyated TCRα and potently stabilized various species reflecting partially deglycosylated TCRα states (+CHO) (Figure 2B, top panel, compare lane 3 to 2). In S1′, epoxomicin significantly and selectively stabilized partially deglycosylated TCRα (+CHO) (Figure 2B, top panel, compare lane 6 to 5). Because full-length glycoslyated TCRα is deglycoslyated in the cytosol, stabilization of the deglycosylated species indicates that the proteasome specifically degrades deglycosylated TCRα in the cytosol. These findings verify the inhibitory activity of epoxomicin in our system and confirm that the proteasome degrades TCRα, but not CTA1, in the cytosol in vivo.

When an untreated P1 was incubated with buffer or CE, no deglycosylated TCRα species were detected in the corresponding S2′ (Figure 2B, top panel, lanes 8 and 9). By contrast, an epoxomicin-treated P1 permits CE-specific release of deglycosylated TCRα species into the S2′ (Figure 2B, top panel, compare lane 11 to 10). Interestingly, preincubation of epoxomicin with the CE did not increase the level of deglycoslyated TCRα species in the S2′ (Figure 2B, top panel, compare lane 13 to 12). These data indicate that membrane-bound, but not cytosol-localized, proteasome regulates degradation of deglycosylated TCRα. Furthermore, an activity in the CE is capable of discharging deglycosylated TCRα into the cytosol in a manner similar to CTA1.

To ensure deglycosylated TCRα species are released from the ER membrane but not from a post-ER compartment such as the Golgi, S2′ was incubated with increasing concentrations of Endoglycosidase H (Endo H), an enzyme that removes carbohydrates from ER-, but not Golgi-localized, glycoproteins. Endo H treatment caused the distinct deglycosylated TCRα species to collapse into a single, fully deglycosylated (-CHO) band (Figure 2C, top panel, compare lanes 3 and 4 to 2), demonstrating that deglycosylated TCRα in S2′ is released from the ER membrane. Additionally, treatment of S2′ with increasing concentrations of peptide-N-glycosidase F (PNGase F), an enzyme that removes all carbohydrates from glycoproteins, also collapsed the various deglycosylated TCRα species into a single band (Figure 2C, top panel, compare lanes 5 and 6 to 2). This finding further confirms that the ladder of bands in S2′ represents various TCRα glycosylation states and not TCRα degradation products.

### p97 plays different roles in release of CTA1 and TCRα into the cytosol

Our findings from the in vitro retro-translocation assay demonstrate that the proteasome degrades only deglycosylated TCRα but not CTA1, consistent with previous results in a cellular context. p97 is considered the major driving force behind extraction of most ERAD substrates from the ER membrane for delivery to the proteasome. As CTA1 is not degraded by the proteasome, two independent laboratories interrogated whether p97 affects CTA1 activity in cells. By expressing mutant forms of p97, Abujarour et al. uncovered a modest disruption in CTA1 activity [13], while Kothe et al. found only a negligible decrease in CTA1 cellular response [14]. Thus the precise function of p97 in CTA1 retro-translocation remains unclear. To resolve this discrepancy, we assessed its role using the vitro toxin release assay.

**Figure 3:**
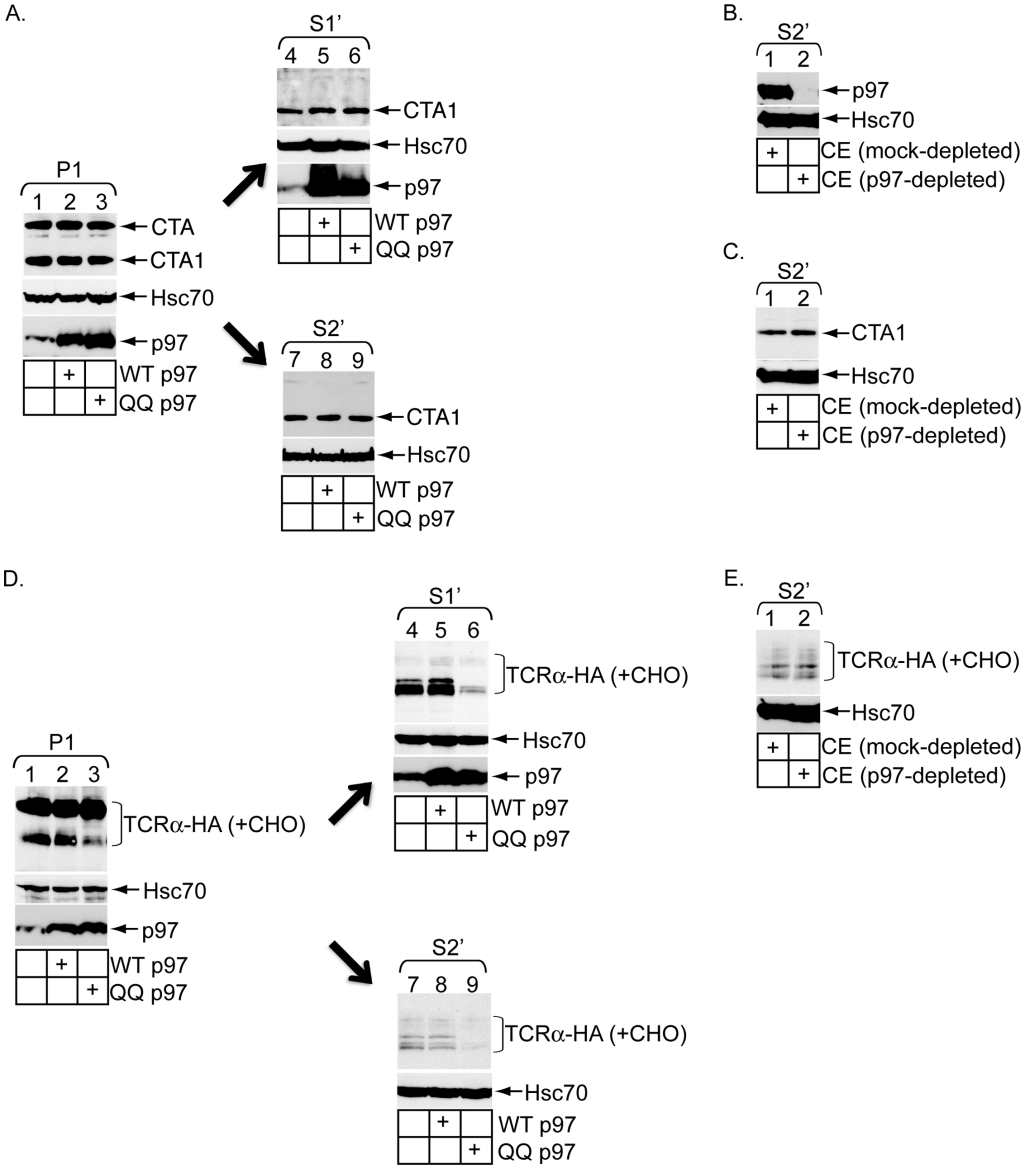
p97 plays different roles in release of CTA1 and TCRα into the cytosol. (A) CT-intoxicated cells transfected with vector, p97-His (WT p97) or p97-QQ-His (QQ p97) were subjected to the in vitro retro-translocation assay. P1, S1′ and S2′ were analyzed by immunoblotting with the indicated antibodies. (B) CE was incubated with a control antibody or antibody against p97. Immune-complexes were precipitated using protein A agarose beads and the resulting supernatants were analyzed by immunoblotting with the indicated antibodies. (C) P1 derived from CT-intoxicated cells was incubated with either mock- or p97-depleted CE. The resulting S2′ was analyzed by immunoblotting with the indicated antibodies. (D) Cells expressing TCRα-HA and co-transfected with vector, WT p97 or QQ p97 were treated with epoxomicin and subjected to the in vitro retro-translocation assay. The resulting P1, S1′ and S2′ were analyzed by immunoblotting with the indicated antibodies. (E) Same as in (C) except P1 was derived from epoxomicin-treated cells expressing TCRα-HA.

Cells transfected with or without WT p97 or the dominant-negative ATPase-defective p97 mutant (i.e. QQ p97) were intoxicated with CT and fractionated to generate P1 and S1′. We found the extent of CTA1 formation in P1 was unaffected by QQ p97 over-expression (Figure 3A, top panel, compare lane 3 to 1 and 2). Additionally, CTA1 appearance in S1′ was also unaffected by this p97 mutant (Figure 3A, top panel, compare lane 6 to 4 and 5). We conclude QQ p97 affects neither CT transport to the ER nor CTA1 retro-translocation in cells. Likewise, the CTA1 level in S2′ derived from QQ p97 P1 was unaffected when compared to the toxin level derived from both untransfected and WT p97 P1 (Figure 3A, top panel, compare lane 9 to 8 and 7). As such, the data suggest that p97 does not stimulate CTA1 release into the cytosol either in vitro or in vivo. To unambiguously prove that p97 does not promote in vitro release of CTA1, p97 was immuno-depleted from the CE (Figure 3B, top panel, compare lane 2 to 1) and the depleted extract tested for its ability to trigger toxin release into S2′. We found that a CE completely devoid of p97 is fully capable of releasing CTA1 into S2′ when compared to a mock-depleted CE (Figure 3C, top panel, compare lane 2 to 1). We conclude that a p97-independent activity drives toxin release from the ER membrane.

We next examined the role of p97 in the release of deglycosylated TCRα. In cells pretreated with epoxomicin, over-expression of QQ p97 decreased levels of deglycosylated TCRα in P1 and S1 in comparison to both over-expression of WT p97 and untransfected control (Figure 3D, top panel, compare lane 3 to 2 and 1, and lane 6 to 5 and 4). Similarly, S2′ derived from QQ p97 over-expressing P1 displayed reduced levels of deglycosylated TCRα when compared to those from untransfected and WT p97 controls (Figure 3D, top panel, compare lane 9 to 8 and 7). Consistent with previous reports, these findings not only demonstrate that QQ p97 over-expression can functionally inactivate retro-translocation, but also indicate an important role of p97 activity in the formation of deglycosylated TCRα in cells [32]–[34]. Surprisingly, when mock- and p97-depleted CE were incubated with an untransfected P1, the levels of deglycosylated TCRα in the resulting S2′ fractions were unaffected (Figure 3E, top panel, compare lane 2 to 1). The simplest explanation for these findings is that, while membrane-associated p97 extracts full-length TCRα from the ER membrane for cytosolic presentation and deglycosylation, additional cytosolic factors acting independently or in cooperation with membrane-bound p97 subsequently discharge the deglycoslyated receptor into the cytosol. The in vitro assay thus reveals that cytosolic events underlying TCRα retro-translocation constitute a multi-step process.

### CTA1 but not TCRα released into the cytosol is largely folded

How does CTA1 avoid p97-dependent targeting to the proteasome for degradation? Using limited proteolysis, an in vitro analysis initially demonstrated that unfolded CTA1 refolds efficiently upon release from purified PDI into bulk solution [10]. Using the same limited proteolysis method and a mutant CTA1, a more recent report suggested that rapid CTA1 refolding in the cytosol in vivo might prevent the toxin from ubiquitination and proteasomal attack [22]. However, these studies did not directly analyze the conformational state of CTA1 in the cytosol. Accordingly, we used limited proteolysis to examine the folding state of in vivo and in vitro retro-translocated CTA1.

**Figure 4:**
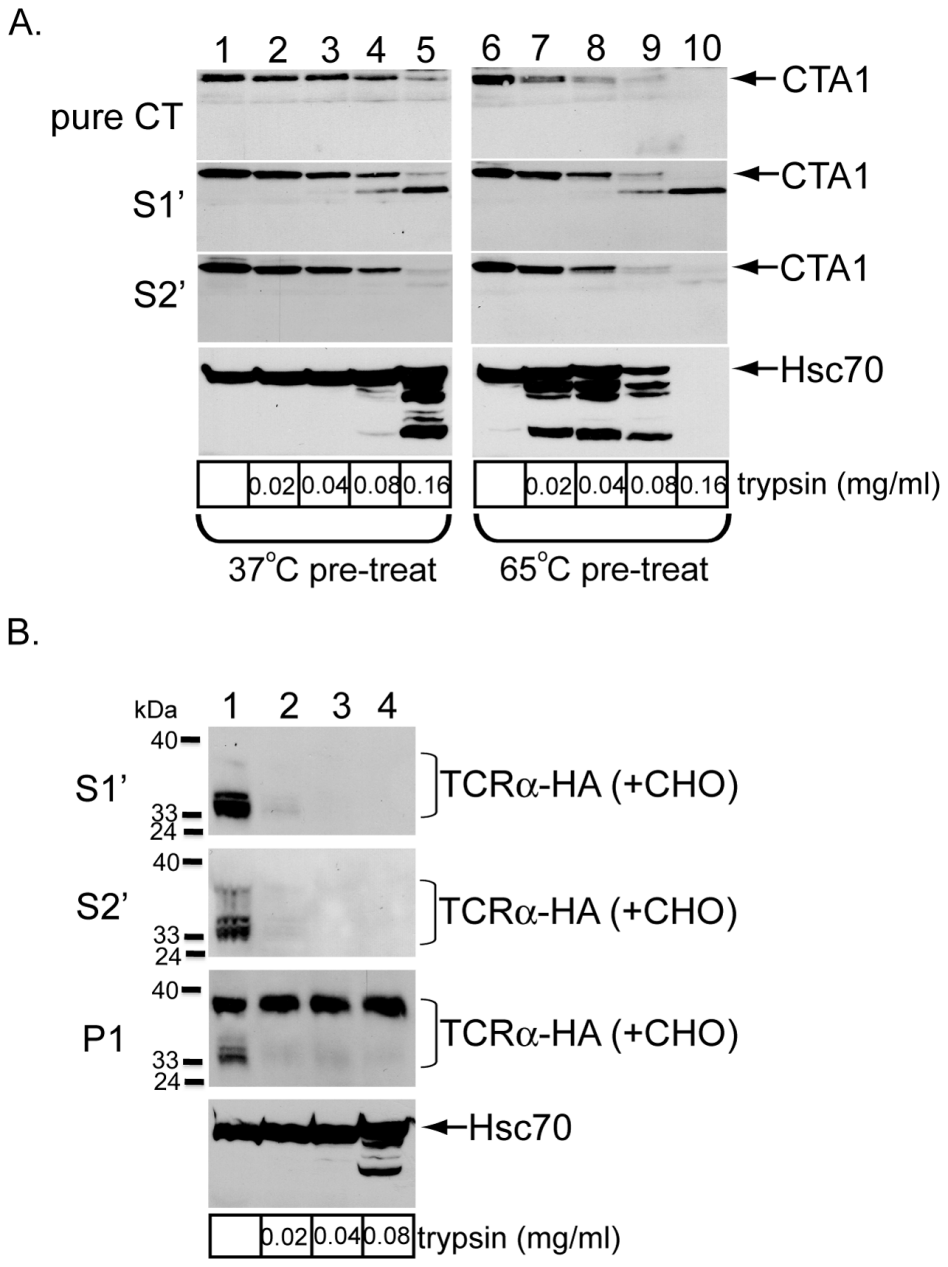
CTA1 but not TCRα released into the cytosol is largely folded. (A) Purified CTA was incubated with CE to mimic the protein concentrations in S1′ and S2′, as well as 20 mM DTT to generate CTA1. S1′ and S2′ containing CTA1 were generated as before. Pure CTA1, S1′, and S2′ were incubated at 37°C (left panels) or 65°C for 15 minutes and then incubated with the indicated trypsin concentration for one hour at 4°C. Reactions were stopped with TLCK and sample buffer and the samples analyzed by immunoblotting with the indicated antibodies. (B) S1′, S2′, and intact P1 were generated from epoxomicin-treated cells expressing TCRα-HA. Samples were incubated with the indicated trypsin concentration for one hour at 4°C. Reactions were stopped and P1 lysed with sample buffer, and the samples analyzed by immunoblotting with the indicated antibodies.

To assess the conformation of CTA1 in S1′ and S2′, we compared sensitivity of the toxin in these fractions to purified, folded CTA. To generate CTA1, purified CTA was incubated with CE (equivalent to the total protein concentrations in S1′ and S2′) and the reductant dithiothreitol (DTT). When purified CTA1, S1′, and S2′ were treated with trypsin, we found that the extent of CTA1 degradation in the three different samples increased similarly with an increase in trypsin concentration (0.02 mg/ml to 0.16 mg/ml) (Figure 4A, top three panels, compare lanes 1–5). In every sample, nearly complete CTA1 degradation was achieved at the highest trypsin concentration (Figure 4A, top three panels, lane 5). When these samples were preheated at 65 degrees, CTA1 became more sensitive to trypsin degradation (Figure 4A, top three panels, compare lanes 6-10 to 1–5). This effect is due to heat-induced CTA1 unfolding, as made evident by the concurrent degradation pattern of the cytosolic chaperone Hsc70. The observation that CTA1 in S1′ and S2′ displayed similar protease sensitivity to each other, as well as to purified CTA1, suggests that the in vivo and in vitro released toxins are largely folded.

In contrast, deglycosylated TCRα in S1′ and S2′ was thoroughly sensitive to trypsin digestion, with the lowest concentration used (i.e. 0.02 mg/ml) essentially degrading all of the receptor (Figure 4B, first and second panels, compare lanes 2–4 to 1). Using the same trypsin concentrations, glycosylated TCRα in intact P1 remained undigested while the deglycoslyated species were sensitive to degradation (Figure 4B, third panel, compare lanes 2–4 to 1). These findings suggest that released, deglycosylated TCRα species in both S1′ and S2′ unlikely refolded or aggregated, but reside in an unfolded conformation for efficient presentation to the proteasome.

### The CTA1 and TCRα release activities do not co-fractionate with established ERAD cytosolic ATPases

**Figure 5:**
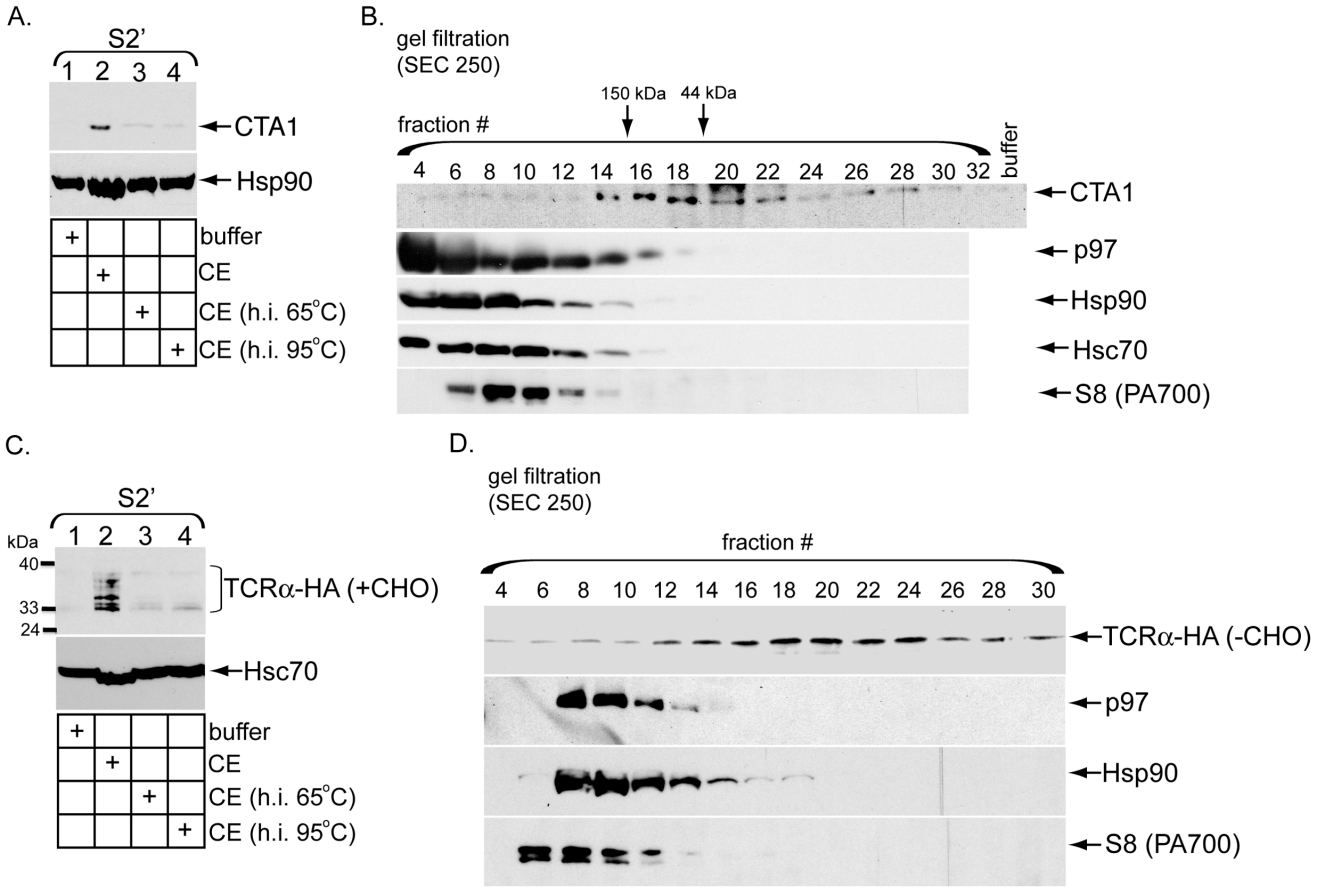
The CTA1 and TCRα release activities do not co-fractionate with established ERAD cytosolic ATPases. (A) P1 from CT-intoxicated cells was incubated with buffer, CE, or CE heat-inactivated (h.i.) at 65 or 95 degrees. The resulting S2′ were analyzed by immunoblotting with the indicated antibodies. (B) 1 ml CE was subjected to size exclusion chromatography by passage through a Bio-Sil SEC 250 column. The indicated even fractions were collected and used to generate S2′ from CT-intoxicated P1. Fractions were also directly analyzed for protein content by immunoblotting with the indicated antibodies (bottom panels). (C) As in A, except P1 derived from epoxomicin-treated cells expressing TCRα-HA was used. (D) 1 ml CE was subjected to size exclusion chromatography by passage through a Bio-Sil SEC 250 column. The indicated odd fractions were collected and used to generate S2′ from P1 derived from epoxomicin-treated cells expressing TCRα-HA. S2′ were treated with PNGase F prior to SDS-PAGE. Fractions were also directly analyzed for protein content by immunoblotting with the indicated antibodies (bottom panels).

We next asked whether the observed cytosolic release activity can be fractionated and, if so, whether it co-fractionates with any ERAD-associated cytosolic ATPases. To initially characterize the cytosolic activity, the CE was heat inactivated at 65 or 95 degrees. Compared to control CE, heat-inactivated CE promoted CTA1 appearance in S2′ less efficiently (Figure 5A, top panel, compare lanes 3 and 4 to 2), further implying that a proteinaceous cytosolic factor induces toxin release. When the CE was fractionated using a gel filtration column, the release activity was observed in fractions corresponding to proteins in the molecular weight range of 44–150 kDa (Figure 5B, top panel, fractions 14–22). p97 and Hsp90, an ATPase hypothesized to mediate CTA1 release into the cytosol [23], do not co-fractionate with the cytosolic release activity (Figure 5B, compare first panel to second and third panels). Fractionation of p97 is consistent with the finding that p97-depleted CE is capable of inducing toxin release (Figure 3C). Furthermore, both Hsc70 and S8 fractionated away from this activity (Figure 5B, compare first panel to fourth and fifth panels). Hsc70 is a cytosolic ATPase implicated in the ERAD process [35]–[37], while S8 is a subunit of the 19S proteasome previously demonstrated to extract substrates from the ER membrane [38]. Thus, the activity responsible for releasing CTA1 into the cytosol is fractionable but does not co-fractionate with any established cytosolic ERAD factors.

Heat inactivation of the CE also blocked its ability to induce appearance of the deglycosylated TCRα species in S2′ (Figure 5C, top panel, compare lanes 3 and 4 to 2). Using gel filtration analyses, the release activity was again shown to fractionate away from p97 (Figure 5D, compare top and second panel), consistent with our previous immuno-depletion results (Figure 3E); the deglycosylated TCRα species appeared as a single band in this experiment due to Endo H treatment. Furthermore, neither Hsp90 nor S8 co-fractionated with the release activity (Figure 5D, compare top panel to third and fourth panels). As with CTA1, we conclude the activity that discharges deglycoslyated TCRα species into the cytosol also represents a unique cytosolic factor.

### Energy-dependent cytosolic activities are required to discharge CTA1 and TCRα into the cytosol

**Figure 6:**
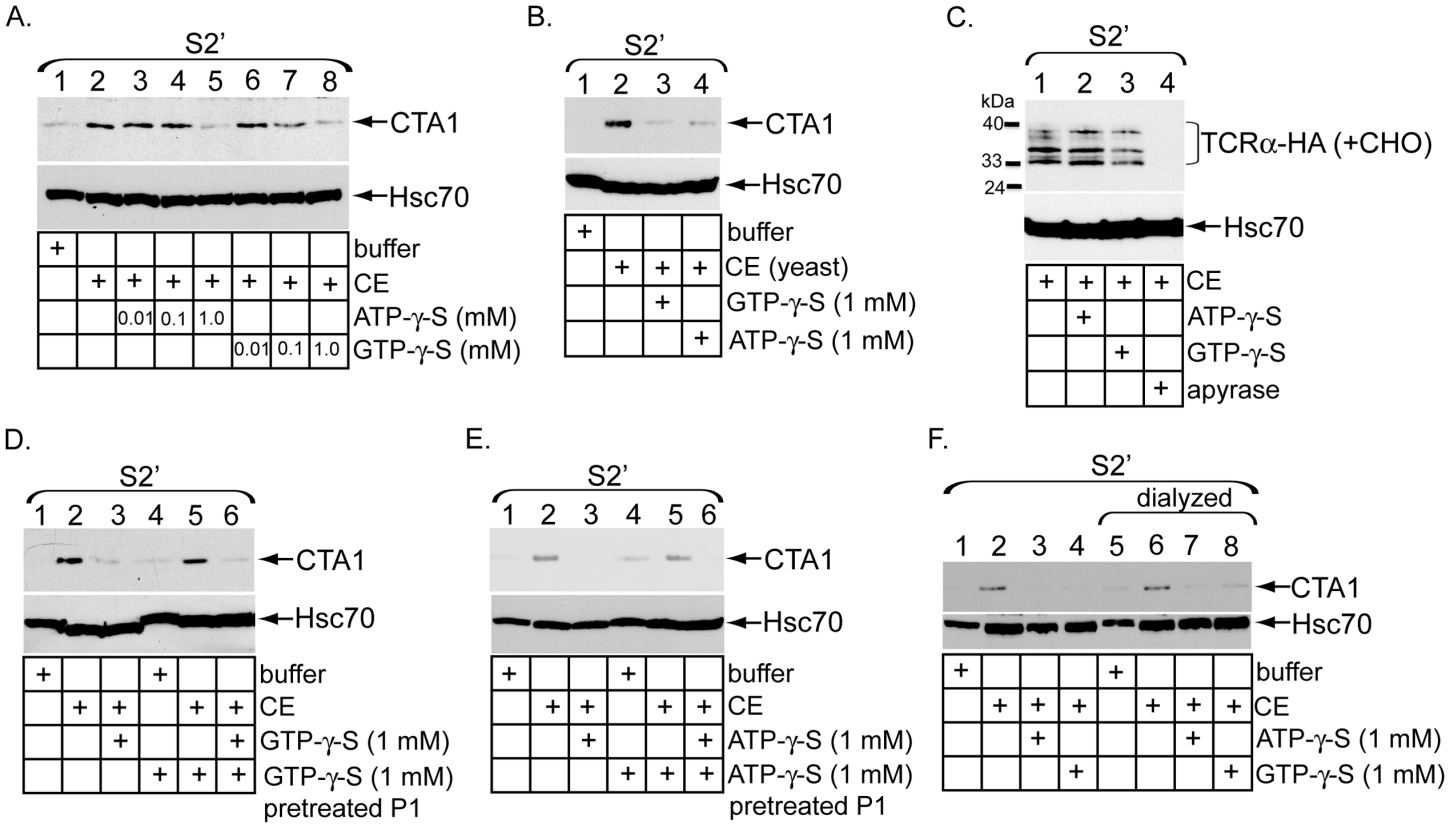
Energy-dependent cytosolic activities are required to discharge CTA1 and TCRα into the cytosol. (A) P1 from intoxicated cells was incubated with buffer, CE, or CE pretreated with the indicated ATP-γ-S or GTP-γ-S concentrations. The resulting S2′ were analyzed by immunoblotting with the indicated antibodies. (B) P1 from CT-intoxicated cells was incubated with buffer, yeast CE, or yeast CE pretreated with 1 mM GTP-γ-S or ATP-γ-S. The resulting S2′ were analyzed by immunoblotting with the indicated antibodies. (C) P1 from epoxomicin-treated cells expressing TCRα-HA was incubated with CE, or CE pretreated with 10 mM ATP-γ-S, 10 mM GTP-γ-S, or apyrase. The resulting S2′ were analyzed by immunoblotting with the indicated antibodies. Identical findings were obtained when 1 mM ATP-γ-S or GTP-γ-S was used (data not shown). (D) CT-intoxicated cells were permeabilized with digitonin buffer with or without 1 mM GTP-γ-S. P1 was washed and incubated with buffer, CE, or CE pretreated with 1 mM GTP-γ-S. The resulting S2′ were analyzed by immunoblotting with the indicated antibodies. (E) As in (D) except with ATP-γ-S. (F) Buffer, CE and CE pretreated with ATP-γ-S or GTP-γ-S were dialyzed 400-fold to remove free nucleotides. Intoxicated P1 were then incubated with undialyzed or dialyzed extracts, as indicated. The resulting S2′ were analyzed by immunoblotting with the indicated antibodies.

To determine if CE-triggered toxin release into the cytosol requires ATP or GTP hydrolysis, S2′ was generated in the presence of increasing concentrations of the non-hydrolyzable ATP analog ATP-γ-S or GTP analog GTP-γ-S. CTA1 appearance in S2′ was significantly attenuated in the presence of either 1 mM ATP-γ-S (Figure 6A, top panel, compare lane 5 to 2) or 1 mM GTP-γ-S (Figure 6A, top panel, compare lane 8 to 2). These findings suggest that both ATP and GTP are required for the release reaction. Interestingly, a CE derived from yeast also induced toxin release into S2′ (Figure 6B, top panel, compare lane 2 to 1), and displayed sensitivity to both 1 mM ATP-γ-S and GTP-γ-S (Figure 6B, compare lanes 3 and 4 to 2). These data indicate that the cytosolic release activity for CTA1 is conserved across species.

We next probed whether ATP-γ-S or GTP-γ-S disrupts release of deglycoslyated TCRα species into the cytosol. In contrast to the toxin release activity, neither ATP-γ-S nor GTP-γ-S perturbed CE-dependent appearance of the deglycoslylated receptor species in S2′ (Figure 6C, top panel, compare lanes 2 and 3 to 1). However, preincubation of the CE with apyrase, an enzyme that hydrolyzes NTP to generate NMP and inorganic phosphates, potently blocked the release reaction (Figure 6C, top panel, compare lane 4 to 1). These data demonstrate that the cytosolic factor responsible for discharging deglycoslylated TCRα species is energy-dependent, although distinct from the release mechanism for CTA1.

To discern whether GTP-γ-S targets a factor in the pellet or cytosol, P1 was generated in the presence of 1 mM GTP-γ-S for 10 min at 4°C, washed to remove excess GTP-γ-S, and incubated with a naïve CE to induce toxin release. In relation to untreated P1, GTP-γ-S pretreated P1 was fully capable of supporting CE-induced CTA1 release into the S2′ (Figure 6D, top panel, compare lane 5 to 2); release was blocked only when fresh GTP-γ-S was added during S2′ generation (Figure 6D, top panel, compare lane 6 to 5). A similar result was observed using ATP-γ-S pretreated P1; release of CTA1 was inhibited only when S2′ was generated in the presence of fresh ATP-γ-S (Figure 6E, top panel, compare lane 5 to 2 and 6 to 5). These results suggest that the putative targets of both GTP-γ-S and ATP-γ-S are likely in the CE. To further examine this possibility buffer, CE, and CE pretreated with ATP-γ-S or GTP-γ-S were subjected to 400-fold dialysis for two hours to remove free nucleotides. Both undialyzed and dialyzed extracts were then incubated with P1 to generate S2′. The results show that dialysis neither compromises the ability of CE to release CTA1 from the membrane (Figure 6F, compare lane 6 to 2) nor reverses the inhibitory effects of ATP-γ-S and GTP-γ-S on the CE (Figure 6F, compare lanes 3 and 4 to 7 and 8). Because the levels of free ATP-γ-S and GTP-γ-S after dialysis are below their inhibitory thresholds (see Figure 6A), the observed block in activity cannot result from disabling of pellet-associated factors (Figure 6F, lanes 7 and 8). In accordance with the previous data, cytosolic factors that mediate CTA1 release must be regulated in an energy-dependent manner.

## Discussion

In this study we developed an in vitro retro-translocation assay performed using semi-permeabilized cells to clarify cytosolic events controlling CTA1 release from the ER membrane into the cytosol, a critical CT intoxication step. We then extended this assay to examine the mechanism by which the canonical ERAD substrate TCRα is discharged from the ER into the cytosol. While semi-permeabilized systems were developed previously to study different aspects of the ERAD process [39]–[42], it has not been exploited to address cytosolic events for any ER-directed toxins. Using this strategy, our findings unveil significant differences in how CTA1 and TCRα are ejected from the ER membrane into the cytosol.

In the case of CTA1, cytosolic release of the toxin does not require p97, a finding consistent with a previous report [14] but in opposition to another [13]. This assertion is based on four independent observations. First, over-expression of a catalytic-inactive p97 did not block toxin retro-translocation in cells. Second, a membrane fraction harboring a catalytic-inactive p97 nonetheless supports CE-induced toxin release into the cytosol. Next, a CE completely devoid of p97 promotes CTA1 release into the cytosol. And finally, fractionation of the CE indicates that the toxin release activity does not overlap with p97-containing fractions. These results convincingly demonstrate that a p97-independent activity is responsible for releasing CTA1 into the cytosol. Because both previous studies examined the role of p97 in intact cells, any disruption of CTA1 retro-translocation can be attributed to upstream functions of p97 including endocytosis [17], [18], Golgi/ER biogenesis [19], and ER fusion [20].

In agreement with previous reports [32]–[34], p97 catalyzes an important step in TCRα retro-translocation and degradation. Specifically, expression of an ATPase-defective p97 mutant decreased appearance of deglycosylated TCRα species in both membrane as well as in vivo and in vitro cytosolic fractions. The simplest interpretation of this finding is that p97 normally extracts TCRα from the ER membrane, exposing the sugar modifications on the receptor to the cytosol for deglycosylation. Our in vitro assay also establishes that deglycosylated TCRα does not fold or aggregate upon release into the cytosol. While the precise mechanism responsible for holding TCRα in the unfolded soluble state is not clear, the cytosolic BAG6 chaperone holdase complex may be involved [12]. Regardless, rendering cytosolic TCRα unfolded is crucial for it to properly engage the proteasome for efficient degradation.

Although the proteasome is primarily localized to the cytosol, a fraction has been shown to interact with the ER membrane [43]. While proteasome-dependent degradation of deglycosylated TCRα aligns with previous findings [30], [31], our data indicate that membrane-associated but not cytosol-localized proteasome is utilized to degrade the receptor. Specifically, stabilization and release of TCRα via pharmacological inhibition of membrane-bound proteasome are not increased by additional inhibition of cytosol-localized proteasome. It is possible that the membrane-bound proteasome pool employs unique adapters to efficiently capture the deglycosylated receptor. Alternatively, membrane-associated proteasome may specifically degrade misfolded membrane substrates while cytosol-localized proteasome degrades soluble substrates. For example, an in vitro assay in yeast showed that cytosol-localized proteasome degrades the soluble ERAD substrate pro-α factor [44]. This does not explain, however, how CTA1 avoids degradation by the proteasome.

Using a protease sensitivity strategy, two independent studies showed that CTA1 refolds rapidly in vitro when unfolded and released from PDI [10], [22]. However, neither report directly probed the conformation of cytosol-localized toxin. By incorporating protease treatment into our assay we have generated the most direct evidence that CTA1 refolds upon release into the cytosol. Whether toxin refolding is spontaneous or is supported by additional cytosolic components is unclear, although lack of CTA1 ubiquitination has been suggested to facilitate toxin refolding [10]. Since substrates normally destined for proteasomal destruction are held in a loosely folded or unfolded state [45], CTA1 refolding not only is necessary for its catalytic function but also offers a mechanism to evade proteasomal degradation. Consistent with previous cell-based findings [10], [21], our in vitro assay demonstrates that the proteasome does not degrade CTA1. The premise that rapid refolding of a retro-translocated toxin can protect against proteasomal destruction is not unique to CT, as the plant toxin ricin also refolds in the cytosol, possibly with the aid of ribosomes [46] and Hsp70 [47].

As discussed in relation to proteasome activity, a unique advantage of the in vitro retro-translocation assay is its ability to uncouple events between the ER membrane and the cytosol; this attribute is most elegantly demonstrated by immuno-depletion of p97. The myriad functions of p97 confound the ability to analyze the cytosolic phase of retro-translocation without provoking pleiotropic effects. Within the parameters of our assay, on the other hand, cytosolic factors can be directly manipulated and reintroduced to a WT membrane fraction. Accordingly, we generated a CE completely devoid of p97 and surprisingly found that the p97-depleted extract was fully capable of inducing TCRα release. Hence, additional cytosolic factors are required to discharge the receptor into the cytosol following p97-dependent extraction from the membrane. These cytosolic factors may act independently or in concert with membrane-associated p97 to catalyze the release reaction. Our assay thus provides the ability to distinguish between membrane-extraction and cytosolic-release of TCRα. Importantly, this observation challenges the conventional model depicting p97 as exclusively responsible for extracting and discharging TCRα into the cytosol. Because p97 is not catalytically involved in CTA1 retro-translocation, it is unclear how the toxin is extracted from the ER membrane and presented for retro-translocation. However, refolding of CTA1 may provide sufficient mechanical energy to extract the toxin from the membrane and prevent backsliding into the ER lumen.

Having effectively eliminated p97, we sought to analyze other prominent cytosolic candidates for the release factor. Using gel filtration analyses we found that the peak toxin and TCRα release activities did not co-fractionate with Hsp90, an ATPase postulated to facilitate toxin release into the cytosol [23]; Hsc70, another ATPase implicated in release of ERAD substrates into the cytosol [35]–[37]; or the19S cap, a proteasomal subunit shown to extract substrates from the ER membrane during ERAD [38], [48]. While both the toxin and TCRα release activities correlate to a molecular weight range of 44–150 kDa, analyses of their energy dependency revealed a final mechanistic difference. CTA1 release is inhibited by binding of ATP-γ-S and GTP-γ-S to soluble factors in the cytosol. In contrast, deglycosylated TCRα release is not sensitive to ATP-γ-S or GTP-γ-S but still depends on energy availability as verified by apyrase treatment. This result emphasizes the utility of our assay at identifying and characterizing divergent ERAD pathways.

Over fifteen years ago, Hazes and Read postulated that many ER-directed toxins like CT, shiga toxin, and ricin appropriate ERAD machinery to gain access to the cytosol and exert cytotoxicity [8]. While these toxins may safely hijack ERAD machinery in the ER to gain cytosol entry, they must deviate from conventional ERAD pathways to evade proteasomal degradation and become cytotoxic. Since the initial proposal, many ER-directed toxins have been shown to co-opt a variety of ER lumenal and membrane components to reach the cytosol [49], [50]. However, cytosolic events remain poorly characterized for these toxins, likely because of the lack of reliable assays to specifically analyze this step. We have presented a simple yet robust in vitro retro-translocation assay that directly addresses how a bacterial toxin and a canonical ERAD substrate are discharged into the cytosol. We anticipate this assay should be easily adapted to study other ER-directed pathogens and endogenous ERAD substrates.

## Supporting Information

Figure S1 **Additional markers to further establish the in vitro cholera toxin retro-translocation assay.** (A) As in Figure 1B, except antibodies against Hsp27 and BiP were used for immunoblotting. (B) As in Figure 1C, except antibodies against Hsp27 and BiP were used for immunoblotting.
(TIF)
