# Supplementary figures and images for: Establishment of an In Vitro Transport Assay That Reveals Mechanistic Differences in Cytosolic Events Controlling Cholera Toxin and T-Cell Receptor α Retro-Translocation
Source: PLoS One. 2013 Oct 11;8(10):e75801. doi: 10.1371/journal.pone.0075801 (PMC3795749; doi:10.1371/journal.pone.0075801)

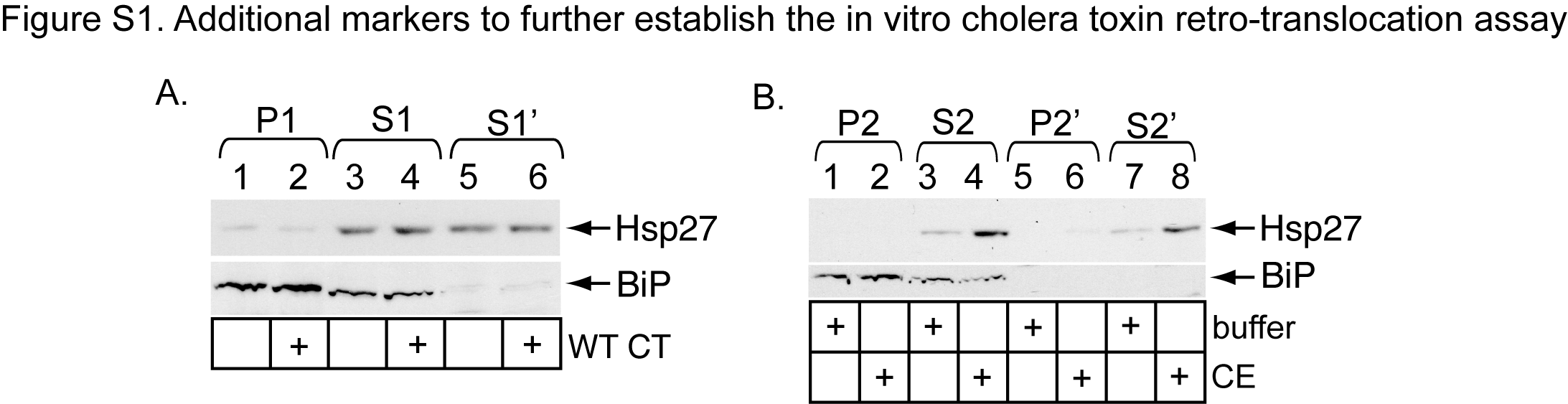

Supplement: Figure S1 — Additional markers to further establish the in vitro cholera toxin retro-translocation assay. (A) As in Figure 1B, except antibodies against Hsp27 and BiP were used for immunoblotting. (B) As in Figure 1C, except antibodies against Hsp27 and BiP were used for immunoblotting. (TIF) [file pone.0075801.s001.tif]
